# Hospital Management and Public Health Role of National Hospitals after Transformation into Independent Administrative Agencies

**DOI:** 10.3390/healthcare10102084

**Published:** 2022-10-19

**Authors:** Yoshiaki Nakagawa, Kaoru Irisa, Yoshinobu Nakagawa, Yasuhiro Kanatani

**Affiliations:** 1 Department of Clinical Pharmacology, School of Medicine, Tokai University, 143 Shimokasuya, Isehara 259-1193, Japan; 2 Department of Respiratorology, Tokyo Medical Center, 2-5-1 Higashigaoka, Meguro-ku, Tokyo 152-8902, Japan; 3 Emeritus President, Shikoku Medical Center for Children and Adults, 2-1-1 Senyu, Zentsuji 765-8507, Japan

**Keywords:** hospital management, financial management, independent administrative agency, infrastructure, health crisis

## Abstract

The development of medical care, technological advances, and the ageing of society have led to rising medical costs. As a result, there is a demand to improve the efficiency of healthcare delivery systems, including public healthcare institutions, in order to ensure the sustainability of healthcare functions. In 2004, as part of national civil service reform in Japan, national hospitals were merged in order to form the National Hospital Organization (NHO). The NHO used new public management methods and was required to be self-financing and to maintain critical functions under a five-year management plan. The objective of this study was to examine whether the NHO was able to maintain its key function in the national infrastructure in terms of management. An analysis of the business conditions of the NHO was performed based on the financial statements from FY 2004 to FY 2018 using evaluation indexes. In the first and second periods, the NHO achieved its targeted management improvements. However, since FY 2014, even with the utmost restrictions on capital investment, the profits have not increased, and the free cash flow has been negative. Our results suggest that further organizational reforms are needed in order to sustain the NHO infrastructure in the long term and to withstand health crisis management during periods such as the COVID-19 pandemic.

## 1. Introduction

Rapid changes in the social environment, i.e., the rapid ageing of society, the changes in the structure of disease, and the advances in medical science and technology, have led to rapidly rising healthcare costs, and the sustainability of healthcare delivery systems has become an issue in Japan and in other countries [1,2,3]. Various hospital reforms have been implemented in Asia, particularly in public hospitals. One of these is the reform of public hospitals using the new public management approach, which has been introduced in Japan and in several other countries [3,4,5]. The largest group of public hospitals that have been reformed using this method in Japan is the National Hospital Organization (NHO), which includes about 140 hospitals.

In 2004, as a part of national civil service reform, the National Hospital Organization (NHO) was formed from national hospitals and sanatoriums in Japan as an independent administrative agency, with the aim of improving the management and the efficiency of medical care through the use of an independent accounting system that is used in most private hospitals [4,5]. The main differences with private hospitals are the management restrictions that are based on salary schedules and staffing, which are based on the standards of the national civil service era; the control of projects that compete with private hospitals, which are considered to put pressure on the private sector; a five-year plan as a medium-term target management corporation; and the operation and management under state supervision based on the plan. In addition, while the government has the authority to assign the President and the Vice-President, the hospital director can appoint the hospital staff with the approval of the President and the Vice-President. The personnel structure is the same as that of the private hospitals in Japan, except that the government appoints the ultimate management head.

In particular, in terms of management, under the Act on General Rules for Independent Administrative Agencies, the NHO is required to formulate a medium-term plan covering five years, to formulate an annual plan that is in line with this medium-term plan, and to manage their operations following the plan that was approved by the competent minister [4,6]. In addition, the NHO is obliged to report their business to the competent minister every fiscal year. Article 15 of the Act on the National Hospital Organization, Independent Administrative Agency stipulates the following four purposes for establishing the NHO and the scope of their operations [6]:

(i) To provide medical care;

(ii) To conduct surveys and research on medical care;

(iii) To provide training for medical technicians;

(iv) To perform services that are incidental to the services that were listed in the preceding three items.

The NHO must fulfil the objectives of the scope of its operations in this law and prepare the mid-term plan and the reports for the mid-term plan. In other words, the management plan that is drawn up is regulated and approved by the government. This includes all of the investment plans and the plans for establishing beds and hospitals; management is carried out in order to fulfil these items. This is a management style that differs significantly from that of private hospitals. The mid-term and annual plans include the following four items, which can be further divided into three to five sub-items [6]:

I. Matters concerning the improvement of the quality of services and other operations that are provided to the public;

II. Matters that are related to the efficiency of business management;

III. Matters that are related to the improvement of the financial status;

IV. Other matters.

The three items that are considered to be within the scope of the NHO operations are listed in (I). These qualities of services are separately monitored as “Clinical Services”, “Clinical Research Business”, and “Education and Training Business” by the government [7,8,9]. This means that, although it is formally independent from the government in terms of its management, its policies as a healthcare organization are always regulated by the government.

Under the national insurance system, the hospital management in Japan is subject to maintenance standards for insurance reimbursement. The only way to reduce management costs is to reduce the portion of the hospital that is not in operation. In particular, the standard number of nurses in hospital wards is strictly regulated according to the amount of reimbursement. At least one nurse must be assigned for 24 h daily for every seven patients in order to receive the highest reimbursement. In other words, if a hospital reduces its staffing too much, it will be audited by the Ministry of Health, Labor and Welfare (MHLW), even if it is a private hospital, and its fees will be refunded, or its license will be revoked. Thus, in Japan, everything is controlled by staffing, and no outcome data have been obtained in order to allow for a comparison of the benefits of treatment. Even in the private sector, the personnel costs are highly constrained, and the personnel cost ratio is the most significant cost item, at around 50%, which is unavoidable under the insurance system. In addition, medical safety issues, such as nosocomial infections, are subject to supervision by the local health department. The establishment permit will be revoked if the hospital does not take action. As has been described above, the medical institutions are treated equally and legally, regardless of their organizational form. The most significant difference is that the management objectives require permission from the minister with the jurisdiction (Table 1).

The independent administrative agency system in Japan was adopted with reference to the agency system in the United Kingdom, with the aim of separating the functions that are related to policy planning from those that are related to the implementation in order to ensure that the implementation functions are carried out efficiently and effectively [10]. However, from its inception, there were major concerns that the Japanese-style independent administrative agency system was a new form of government corporation that would lead to strengthened political control [11,12].

**Figure 1:**
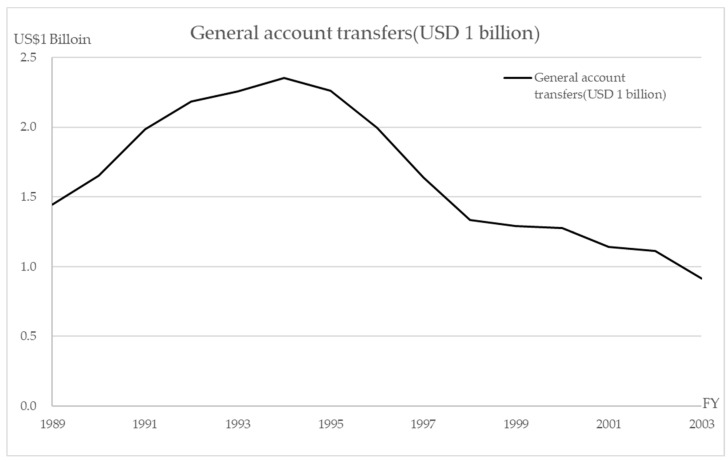
General account transfers (USD 1 billion).

The most important reason for the reorganization into an independent administrative agency was to reduce or to eliminate the transfer of funds from the national budget to the national hospitals [5,10]. For example, as shown in Figure 1, about USD 1 billion (USD 1 = JPY 110) was transferred from the national general account in 2003 and about USD 2.35 billion at the peak in 1994 [13,14,15,16]. Therefore, improving the management and the efficiency of medical care (i.e., increasing healthcare “sales” and operating on a low budget) became the most important mission of the NHO. The NHO achieved a surplus for five consecutive years by restricting the new investment and securing the human resources, with the aim of increasing the medical treatment profit in the first five-year period (April 2004 to March 2009) [17]. In the second period, the investment was gradually resumed, with a further reduction in hospital beds and attempts to reduce the costs of materials, etc.; the current account balance exceeded 100% each year [4,18]. In the third period, increasing the number of medical staff in order to obtain higher reimbursement without changing the business structure led to an imbalance between the labor costs and the medical business income gradually becoming apparent. These results were evaluated by the Independent Administrative Institution System Evaluation Committee and were published as evaluation results by the competent minister, the Minister of Health, Labor and Welfare [7,8,9]. As a result, the “III. Matters Related to Improvement of Financial Condition” was rated “S” (much above target) or “A” (target achieved) in the first and second periods, but “B” (treated as achieved but some targets did not achieve) in the third period. To the best of our knowledge, no further detailed investigation of these issues has been conducted. In this study, we focused our analysis and review on the financial relations that were rated the final score of “B” in the third period. (Table 2).

The NHO hospitals provide medical care from the chronic to the acute phases and can be considered to be a microcosm of the Japanese healthcare system. The management analysis in this study was conducted by classifying the NHO by healthcare function in order to identify the managerial problems in the healthcare system in Japan. In addition, the NHO has an important function in the national infrastructure for health risk management, such as that required in the event of a pandemic, such as COVID-19, or in a large-scale disaster, such as the 2011 great East Japan earthquake [18]. This function is a permanent role that has to be fulfilled in the long term as infrastructure. On the other hand, if the NHO is to become operationally independent, the most important issue is its potential viability in terms of the balance of payments. Therefore, in this study, the management status of each NHO hospital group has been analyzed using an original index in order to examine the problems that were brought about by the efficiency improvement of medical management after the establishment of the NHO as an independent administrative agency.

## 2. Materials and Methods

The study covered three periods (15 years) from April 2004 to March 2019. The business conditions were analyzed for each period by using financial statements that were published by the NHO, especially the profit and loss (PLS) and the cash flow statement (CFS) [19]. The items used in the PLS were limited to the medical service revenue and medical service cost portions. The CFS was not categorized by business, therefore, the entire cash flow was used. The financial statements were for 142 hospitals (141 in FY 2018, after one hospital was discontinued). For the analysis, these hospitals were categorized into six groups, using the NHO classification, as follows: hospitals offering acute-phase medical care with <349 beds (group 1), 350–499 beds (group 2), and >500 beds (group 3); hospitals offering medical care for disabilities (group 4); hospitals offering psychiatric medical care (group 5); and hospitals offering mixed medical care (group 6).

The following indicators were used in the analysis. From the PLS, the rate of increase in the medical revenue was based on the revenue that was shown in the medical service section. The growth rate was obtained by calculating the medical revenue per hospital bed for each hospital and then calculating the average for each group by determining the difference from the previous year’s figures and dividing by the amount of the previous year. The same method was used for the growth rate in the previous year. The rate of increase in each group was calculated as the rate of increase = (average value per hospital bed − average value per hospital bed in the previous fiscal year)/average value per hospital bed in the previous fiscal year × 100%. The rates of increase in personnel-related expenses, material costs, and capital investment expenses were obtained using the salaries and outsourcing costs, material costs, and equipment-related costs, respectively, in the medical service costs. The five-year averages were calculated for each group for the growth rates of the medical revenues, personnel-related expenses, material expenses, and equipment-related expenses.

**Figure 2:**
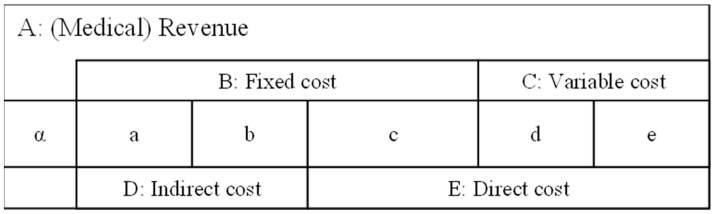
PL structure A: (Medical) Revenue, B: Fixed cost, C: Variable cost, D: Indirect cost, E: Direct cost, α: Medical profit, a: Depreciation for a hospital, b: Maintenance cost for a hospital, c: Labor costs, d: Cost of medical materials, e: Food expenses. A = α + B + C = α + D + E, B = a + b + c, C = d + e, D = a + b, E = c + d + e.

The management index that we developed in 2010 was used for the analysis of the PLS. In this approach, the ratio of marginal profit after personnel cost to personnel cost (RMP), the ratio of investment per personnel cost (RIP), and the operating profit per personnel cost (OPP) were converted into USD 1 of labor cost [17,20,21]. These indicators can be expressed as follows from the relationship between the cost of medical expenses and medical income A in the PLS of the hospital shown in Figure 2:

Indicator 1: Ratio of the marginal profit after personnel cost per personnel cost (RMP), as follows: $RMP=\frac{A-E}{c}=\frac{\alpha +a+b}{c}$
Indicator 2: Ratio of investment (= indirect cost) per personnel cost (RIP), as follows: $RIP=\frac{D}{c}$
Indicator 3: Operation profit per USD 1 of personnel cost (OPP) (difference between the RMP and the RIP), follows: $OPP=\frac{\alpha }{c}=RMP-RIP$
The indicator OPP represents the efficiency of medical management.

These indicators are generally expressed in relation to labor costs, which are the largest cost item in Japanese healthcare, accounting for around 50% of costs, and can be used for management benchmarking between healthcare organizations [20,21]. 

First, the break-even point (BEP) is α = 0, i.e., zero medical profit. In other words, when the OPP = α = 0, from the OPP formula, RIP = RMP is the BEP. This means that the BEP can also be determined using the following formula [20,21]:

$$\begin{array}{cc} BEP & =\frac{Fixed\;cost}{1\;-\;\left(\frac{Variable\;cost}{Revenue}\right)}=\frac{B}{1\;-\;\frac{C}{A}}=\frac{a\;+\;b\;+\;c}{\frac{A\;-\;C}{A}}=\frac{A\left(a\;+\;b\;+\;c\right)}{\alpha \;+\;a\;+\;b\;+\;c}=\frac{A\left[c\left(\frac{a\;+\;b}{c}\right)\;+\;c\right]}{c\left[\left(\frac{\alpha \;+\;a\;+\;b}{c}\right)\;+\;1\right]}=\frac{A\left(c\;\times \;RIP\;+\;c\right)}{c\left(RMP\;+\;1\right)}\\ & =\frac{Ac\left(RIP\;+\;1\right)}{c\left(RMP\;+\;1\right)}=\frac{A\left(RIP\;+\;1\right)}{RMP\;+\;1} \end{array}$$

Then, using the indicators RMP, RIP, and BEP, the break-even ratio (BER) can be expressed as follows:

$$BER=BEP\times \frac{1}{A}=\frac{A\left(RIP+1\right)}{RMP+1}\times \frac{1}{A}=\frac{RIP+1}{RMP+1}$$

$$BER\left(\%\right)=BER\times 100\left(\%\right)=\frac{RIP+1}{RMP+1}\times 100\left(\%\right)$$

The relationship between the RMP and the RIP can also be expressed using the BEP, as follows: $\mathrm{RIP}=\mathrm{BEP}\left(\mathrm{RMP}+1\right)-1$

**Figure 3:**
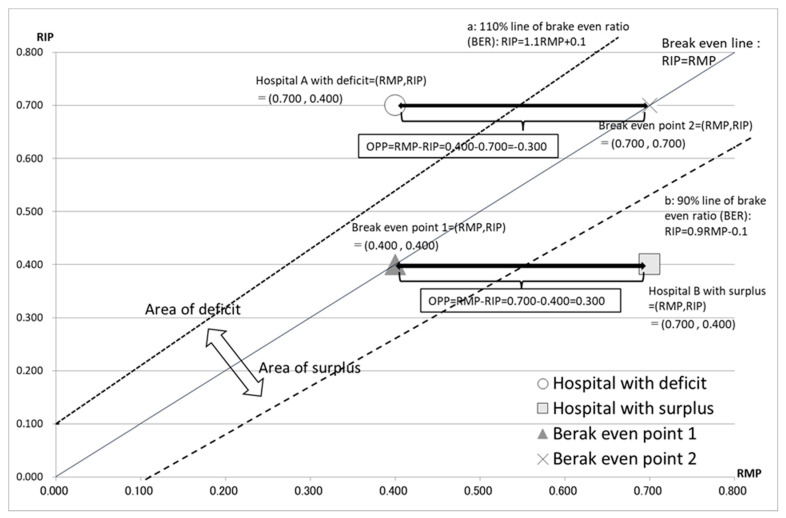
The relationship between RMP, RIP, OPP, and the break-even line. Hospital A, with a deficit, is shown as (RMP, RIP) = (0.400, 0.700). Hospital B, with a surplus, is shown as (RMP, RIP) = (0.700, 0.400). The line RIP = RMP is the break-even line. Line “a” represents 110% of the break-even ratio (BER): RIP = 1.1RMP + 0.1, calculated by the formula: RIP = BER (RMP + 1) − 1. Line “b” represents 90% of the BER: RIP = 0.9RMP − 0.1. The width between hospital A and BEP of 0.400 − 0.700 = −0.300 shows the operating profit per USD 1 of personnel cost (OPP) of hospital A. The width between hospital B and BEP of 0.700 − 0.400 = 0.300 shows the operating profit per USD 1 of personnel cost (OPP) of hospital B. The area over the BER line is the deficit area and that below the BER line is the surplus area.

The relationship between the RMP, the RIP, the OPP, and the break-even line is shown in Figure 3. 

Using the CFS, the free cash flow (FCF) was obtained from the sum of the cash flow (CF) from the operating activities and investment activities, and the average value over five years was calculated for each group.

## 3. Results

### 3.1. Medical Revenue

The medical revenues increased from 1.1% to 2.9% in the third five-year period but were 1–2% lower than in the first and second periods (Table 3). The increases were lowest in 2016, at <1%, except in groups 1 and 6. These groups increased by 3.3% and 3.2% in 2016, respectively; however, these values were not reflected in the RMP. This suggests an increase in the labor costs beyond the increase in the medical revenues. In the third period, the increase for all of the NHO hospitals was 1.9%, and the medical revenues in the acute care groups 1, 2, and 3 increased by >2%.

### 3.2. Personnel-Related Expenses

There were very few years in which the expenses that were related to labor costs decreased (Table 3). The only decreases were in group 4 by 3.1% in 2006, in group 5 by 0.2% in 2008, and in group 6 by 1% in 2005 and by 3.2% in 2018. The acute care groups 1 to 3 had increased personnel expenses in all of the years. The overall average increase over the 15 years was 3.3% and was almost the same for the three five-year periods. The highest increase of 7.0% occurred in 2015 and the highest group increase of 9.2% occurred in group 2.

### 3.3. Material Costs

The average cost of the medical materials for all of the NHO hospitals increased in all periods (Table 3). However, the rate of growth was lower in the third period compared to the first and second periods. In the third period, the rate of increase was <3%, except in 2015, but groups 2, 3, and 6, which provide acute care, showed large increases of 4.9%, 4.2%, and 3.6%, respectively. Group 3, in particular, did not have a single year of decline in 15 years. However, since 2016, the growth rate of material costs has been <3%.

### 3.4. Capital Investment Expenses

**Figure 4:**
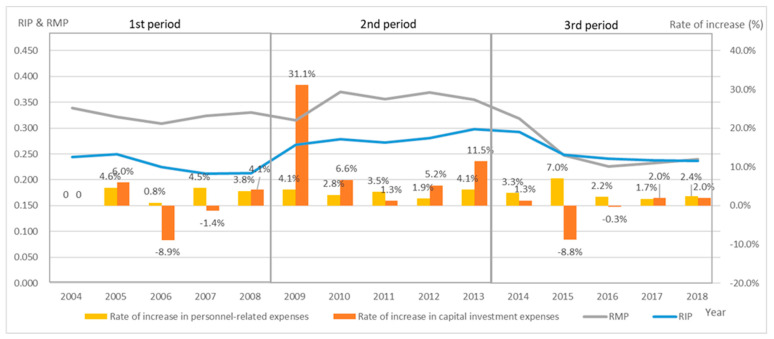
Relationship between RIP and the rate of capital investment expenses of all NHO hospitals. The RIP remained high, above 0.25, for the second period from FY 2009 to FY 2014. This indicates that the NHO had sustained investment during this period. The rate of change, which shows the rate of growth from the previous year, showed an increase of over 30% in FY 2009 and sustained that investment until FY 2014; the investment was restrained in the third period from FY 2015 onwards.

As shown for the RIP (Figure 4), little capital investment was made in the first period, but this increased by 11.1% in the second period (Table 3). This impact continued until 2014. The RMP also increased from FY 2010, but this was presumably due to the effects of capital and personnel investment. In the third period, investment was curtailed again from 2015, which was triggered by increased labor costs in 2015, and both the RIP and the RMP decreased. These effects continued until the end of the third period. There was a significant decline in all of the groups in 2015, with an average of −8.8% for the year, and the impact is shown in the subsequent changes in the RIP. 

### 3.5. RMP, RIP, OPP, and BER

**Figure 5:**
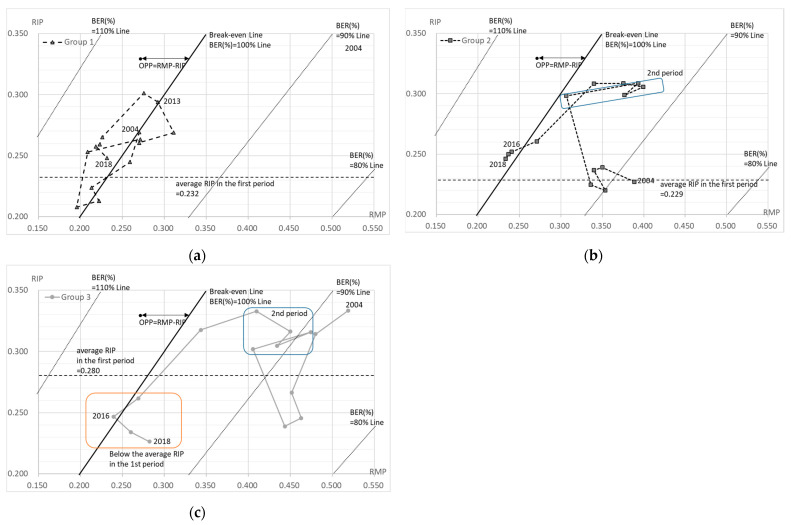
RMP, RIP, OPP, and BER in hospitals offering acute-phase medical care. (**a**) Group 1 (<350 beds); (**b**) group 2 (350–499 beds); (**c**) group 3 (>500 beds). The OPP shows the difference between RMP and RIP; OPP = RMP − RIP. Break-even Line; RIP = RMP. 90% of break-even ratio (BER (%)): RIP = 0.9RMP − 0.1, calculated by RIP = BER (RMP + 1) − 1. 80% of BER (%): RIP = 0.8RMP − 0.20. 110% of BER (%): RIP = 1.1RMP + 0.10.

**Figure 6:**
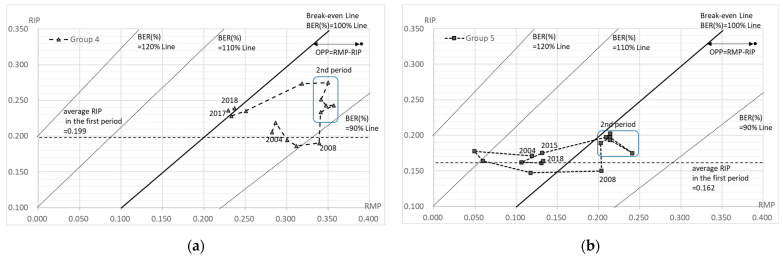

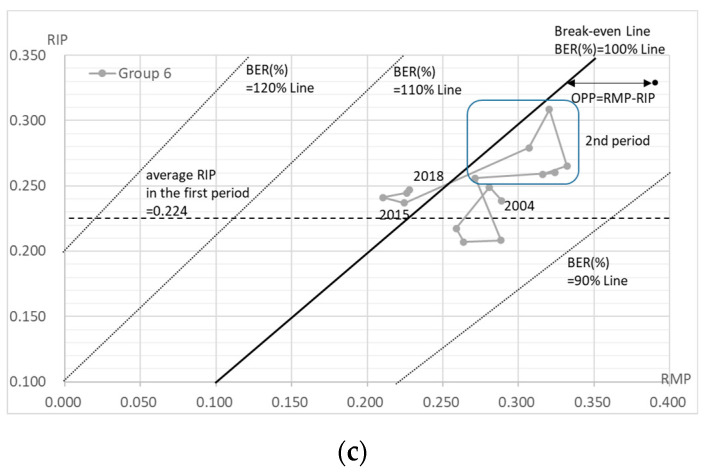
RMP, RIP, OPP, and BER in hospitals offering chronic-phase medical care. (**a**) Group 4 (hospitals offering medical care for disabilities); (**b**) group 5 (hospitals offering psychiatric medical care); (**c**) group 6 (hospitals offering mixed medical care). The OPP shows the difference between RMP and RIP; OPP = RMP − RIP. Break-even Line; RIP = RMP. 90% line of break-even ratio (BER (%)): RIP = 0.9RMP − 0.1, calculated by the formula: RIP = BER (RMP + 1) − 1. 110% line of BER (%): RIP = 1.1RMP + 0.10. 120% line of BER (%): RIP = 1.2RMP + 0.20.

Figure 5 and Figure 6 demonstrate the relationship between the RIP, the RMP, and the rate of increase in the personnel-related expenses in each group. 

Group 1 includes the acute-phase medical care hospitals with less than 350 beds. In the first period, the investment was restrained and both the RIP and the RMP decreased in line with the growth in labor costs (Figure 5a). In the second phase, significant capital investment was made in 2009, and this trend continued throughout the second period (Table 3). Both the RIP and the RMP increased accordingly, showing the effects of the investment. In the third period, the capital investment was restrained, but the impact of increased capital investment in the second period continued after 2014 and the RIP remained high in 2018. On the other hand, the labor costs increased in all periods, especially in FY 2015 in the third period, which increased the gap between the RIP and the RMP (increasing the deficit).

Group 2 includes the acute-phase medical care hospitals with 350–499 beds. The increase in the capital investment started in the first period, with a particularly large increase in capital investment in 2009 in the second period, and continued until 2014 in the third period (Table 3). The labor costs increased during the whole period and, finally, in the third period both the RIP and the RMP decreased at the same time, in line with the growth in labor costs. The capital investment was strongly reduced in FY 2015, but the capital investment from the second period and the continuous increase in the labor costs could not be absorbed, resulting in a deficit (negative OPP) from FY 2016 (Figure 5b).

A clear difference between the first, second, and third periods is apparent in the acute groups since group 1 has had a negative OPP (deficit) since FY 2013 and group 2 since FY 2016 (Figure 5a,b).

Group 3 includes the acute-phase medical care hospitals with more than 500 beds. In the first period, the capital investment was restrained and both the RIP and the RMP decreased in line with the growth in labor costs (Table 3, Figure 5c). In the second period, a significant capital investment was made in 2009, which continued throughout the second period, and the RIP also increased. After the significant capital investment in FY 2009, the RMP increased significantly from FY 2010 and remained high until FY 2013. In the third period, the capital investment became more restrained from FY 2015, while the labor costs continued to increase, resulting in a decline in both the RIP and the RMP, but the labor costs grew significantly in FY 2015 and the OPP became negative (deficit) for the first time in FY 2016. However, the RMP subsequently increased and was above 0.25, resulting in a positive (surplus) OPP. Thus, group 3 increased its RIP in the second period due to capital investment, but this was balanced by high RMP being maintained. However, its RMP declined sharply in the third period, and it was in deficit (negative OPP) for the first time in FY 2016 (Figure 5c). 

Group 4 includes the hospitals offering medical care for disabilities. In the first period, the capital investment was restrained, except in 2005 (Table 3). The labor costs were also restrained, with little increase, except in 2005 (Table 3). As a result, the OPP was positive (surplus). In the second period, a significant capital investment was made in 2009, and this trend continued until 2014 in the third period (Figure 6a). Along with this, the RIP also increased and had an impact until FY 2018. The RMP continuously showed high values, but both the RIP and the RMP had started to decrease in line with the increased labor costs and the decreased capital investment in FY 2015. In the third period, both the RIP and the RMP were relatively stable; however, from FY 2017 they were in deficit (negative OPP).

Group 5 includes the hospitals offering psychiatric medical care. In the first phase, significant personnel and capital investment were made in 2005, but the capital investment was subsequently reduced (Table 3). On the other hand, the investment in labor costs continued during the whole period (Table 3). The business turned into a surplus in 2008, and a significant capital investment was made again in 2009 (Figure 6b). The capital investment was temporarily curbed in 2011, but the RIP also rose again as a result of the increased capital investment in 2012 and 2013. In the third period, while there was a significant increase in human rights expenditure in 2015, the capital investment remained reduced or restrained from 2015, and the RIP decreased. However, the RMP decreased further, causing a significant deficit (negative OPP) to continue from 2015.

Group 6 includes the hospitals offering mixed medical care. In the first phase, both the capital investment and the labor costs were controlled until 2008, and the RIP continued to decline, while the RMP was maintained or showed an upward trend (Table 3, Figure 6c). Both the capital investment and the labor costs increased from 2008, with a significant capital investment in 2009, which was a trend that continued throughout the second period. The RIP increased accordingly, and its impact continued until FY 2018. The RMP has been high since 2010, but the RMP decreased sharply in line with the increase in labor costs in 2015. The impact of increased labor costs was observed over the entire third period, with a deficit (negative OPP) from FY 2015.

**Figure 7:**
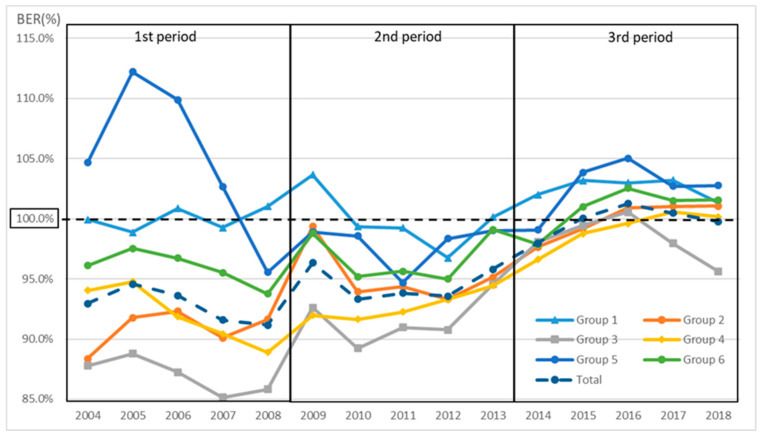
Calculation of BER (%) for each group. BER (%) above 100% means a deficit and a BER (%) below 100% means a surplus; in 2017 and 2018, all but group 3 were in deficit.

The BER (%) can also be read in Figure 5 and Figure 6. It is shown as the range of each BEP, which is indicated by RIP=BEP (RMP+1) −1. Furthermore, Figure 7 shows the BER (%) for each group for each year, where a BER (%) above 100% means a deficit and a BER (%) below 100% means a surplus. Period one was the best situation in total, although the second period was seen to be in a better business position. In total, the BER (%) was worse in periods one, two, and three. In addition, all but group 3 were in the red in FY 2017 and FY 2018.

**Figure 8:**
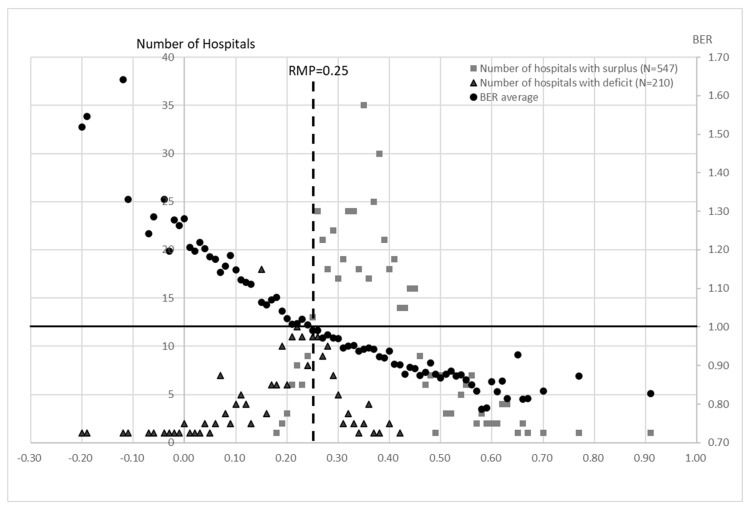
Calculation of BER for each RMP in groups 1, 2, and 3 for all 15 years. The BER shows the average of all hospitals with each RMP value. The number of deficit and surplus hospitals for all years was disaggregated by the RMP value. Most surplus hospitals were located above RMP = 0.25, while most deficit hospitals were located below 0.25. On the other hand, the average BER crosses the line of RMP = 0.25 between surplus and deficit hospitals. This indicates that RMP = 0.25 is a critical point in terms of management.

**Figure 9:**
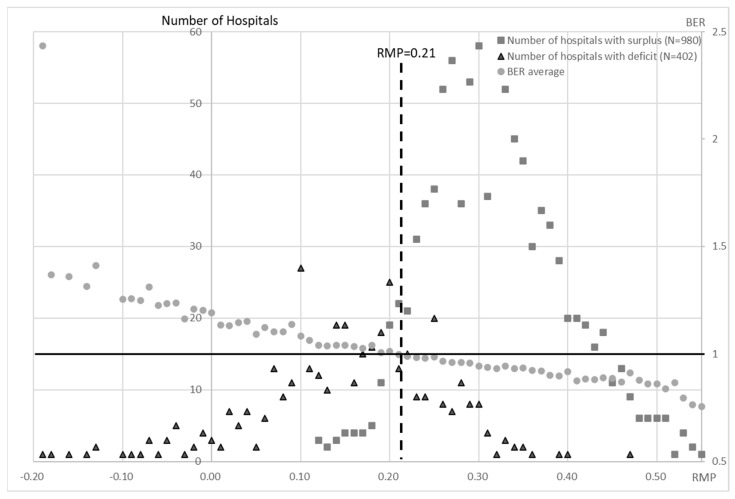
Calculation of BER for each RMP in groups 4, 5, and 6 for all 15 years. The number of deficit and surplus hospitals for all years was disaggregated by RMP value. The BER shows the average of all hospitals with each RMP value. Most surplus hospitals were located above RMP = 0.25, while most deficit hospitals were located below 0.21. On the other hand, the average BER crosses the line of RMP = 0.21 between surplus and deficit hospitals. This indicates that RMP = 0.21 is a critical point in terms of management.

The BER was calculated for each RMP for the acute care hospitals and the chronic care hospitals (Figure 8 and Figure 9). The BER decreased as the RMP increased. The number of hospitals that were in surplus was higher than the number that were in deficit when the RMP was ≥0.25 for the acute hospitals and ≥0.21 for the chronic hospitals (Figure 8 and Figure 9).

### 3.6. Free Cash Flow (FCF)

In terms of FCF, only group 3 had positive averages for all of the three periods, amounting to approximately USD 112.8 million, USD 107.3 million, and USD 48.9 million, respectively. In particular, only group 3 was positive in the third period and was negative only in FY 2016 (Table 3). However, the total for all of the NHO hospitals was negative for the fifth consecutive year, since 2014. The situation was particularly bad for group 1, which was negative in all but FY 2005, FY 2006, FY 2007, FY 2016, and FY 2018. Groups 5 and 6 had negative results for all five years of the most recent period, with five-year averages of approximately USD −16.3 million and USD −60.7 million, respectively.

### 3.7. Number of Hospital Beds

After the formation of the NHO, the number of hospital beds has been steadily reduced in order to decrease the fixed costs and secure profits. The average numbers of beds were 54,359, 52,159, and 51,172 in the first, second, and third five-year periods, respectively, showing a decrease of about 3200 from the first to the third periods (Table 3).

## 4. Discussion

The healthcare system in Japan is characterized by universal health coverage, in which the price of the medical treatment is uniformly determined by the official prices, and, in principle, medical care is provided to the patients in kind [1,2]. At the same time, the source of a hospital’s income from reimbursement is not entirely from insurance premiums, but from the national treasury, insurance premiums, and approximately 30% of the patient’s co-payment [2]. In addition, there is no distinction between public and private hospitals, including the NHO hospitals, in terms of hospital medical income and the competitive drive to recruit more patients, and the reimbursement is balanced against the amount of capital investment [22]. Therefore, in order to maintain good hospital management, there is a tendency to increase the hospital functions and to prioritize the availability of the medical services that can provide a high number of patients and a high unit cost per treatment in order to ensure a profit margin. 

As part of the monitoring of such services, the NHO has created and started using clinical evaluation indicators since FY 2006 for the purpose of evaluating the quality of medical care. Ver. one (FY2006–FY2009) introduced 26 indicators, which were revised every few years, and Ver. two (FY2010–FY2014) used 87 indicators (including 63 process indicators and 7 outcome indicators), while Ver. three (FY2015–FY2010) used 115 indicators (including 102 process indicators and 13 outcome indicators). The Ministry of Health, Labor and Welfare (MHLW) launched a research project known as the “Project to Promote Evaluation and Publication of the Quality of Medical Care” in FY 2010, with the aim of improving the quality of medical care, and it is accepting applications from all over Japan. The project aims to visualize the medical care that is provided by each hospital and to equalize and improve the quality of medical care. However, most of the indicators are process indicators and are not outcome indicators corresponding to the process; they have not yet reached the point where they can be used to evaluate performance.

The other clinical evaluation indicator is an evaluation indicator of business performance that is in line with the mid-term goals. In regard to the evaluation of the mid-term plan, the first to the third period is reported to the minister of MHLW and is rated on five levels of “S”, “A”, “B”, “C”, and “D”, with “S” being the highest rating. The evaluation results show that, except for an increase in the number of items that were rated as “B”, i.e., “treated as achieved but some targets did not achieve” in the third period, the results were rated as “S” or “A” as “significantly above” or “above target”, respectively [7,8,9]. As a result, the agency is rated as “treated as achieved but some targets did not achieve ” in the third period, but the reality is that the number of items that can be monitored numerically for medical functions did not fall much lower than that in the second period, but the increase disappeared (Table 2).

Medical reimbursement is defined in detail in all of the areas by each activity function and, as mentioned above, the conditions of provision are described for each item. However, the healthcare organizations themselves do not conduct the audits or the monitoring of the treatment outcomes that are related to benefits. On the other hand, the number of patients, the state of the medical equipment, and the specialist index, which was removed the advertising restrictions that are set by the MHLW, are published on a commercial basis. The healthcare system in Japan allows patients to visit any hospital if they wish. We have the highest number of beds and hospitals in the world, the management of hospitals is constantly in a competitive environment, and it is highly necessary to keep the service environment up-to-date in order to continue earning high profits. Therefore, the more proactive healthcare organizations will be more attentive to these commercial factors, while focusing their investments on areas where they can earn higher profits more efficiently. 

Furthermore, the medical reimbursements, which define the price for the services and the delivery system, are revised every two years, with various conditions being removed or added repeatedly in order to suit the situation [1,23]. With the system that is related to the ‘medical fee schedule’ as the key in Japan’s healthcare policy, the healthcare delivery environment, which is mostly private, is constantly being induced to be cheaper and maintained at a higher level. As a result of these policy inducements, while the healthcare expenditure as a percentage of GDP is in the top group of OECD countries at around 11%, per capita the healthcare expenditure is not high, and Japan has maintained health outcomes that make it the country with the longest life expectancy in the world [24,25]. This also indicates that the policy has induced the continuous provision of healthcare of a certain level of quality. Furthermore, this approach means that healthcare that has little or no track record of this provision will not be reimbursed itself, inducing voluntary downsizing of functions and concentrating resources in the more general and high-demanded areas. In other words, it can be considered a method of inducing efficiency gains in the healthcare market. The NHO has operated completely independently in such a market environment, competing with the private sector, and not relying on government funding. However, there is a general reluctance to allow public hospitals, including the independent administrative agencies, to strengthen or enter into businesses that compete with the private sector for managerial advantages, as this would put pressure on the private sector businesses. 

The analysis of the 15 years of management performance in this paper provides a basis for determining if public hospitals, which are expected to serve as infrastructure for managing healthcare crises, are financially sustainable after their conversion into independent administrative agencies that are required to have the same financial independence as the private sector while maintaining their current roles. The medical fees are set uniformly in Japan by the government according to the quality and the difficulty of the treatment, and the aggregate medical profit can be viewed as a measure of hospital management. The NHO, similar to the national government, has a common set of salaries according to the job type and age, which makes the comparison with the labor costs a good indicator to benchmark a hospital’s performance [20]. Furthermore, given the staffing and the functional requirements that are linked to the ‘medical fee schedule’, as described above, the costs that can realistically be managed by the healthcare providers are limited to how many personnel are added from the minimum number that is determined in the system, and to what extent the facilities are renovated or the medical equipment is replaced. On the revenue side, the real issue is the balance of provision, i.e., what medical specialties of doctors are employed and what type of medical care is provided. Thus, the managers in Japan only have control over the growth of the labor costs, the amount of investment, and the number of patients, which are closely linked to the corresponding sales. Therefore, the RMP, the RIP and the OPP are effective and simple for the assessment of hospital management in the NHO, in terms of the balance among the medical income, the labor costs, and the medical functions that are associated with the equipment.

The RIP shows the ratio of the indirect costs (investment) to the labor costs, and, thus, indicates the balance between the capital and the labor costs that are required in order to provide medical functions. For example, the new capital expenditure in the third period was on a downward trend, as evidenced by the growth rate of the capital expenditure (Table 3, Figure 4). However, this value was higher in the third period than in the first period, when the capital investment was also limited, due to the impact of capital investment that was made in the second period. On the other hand, the capital expenditure in the PLS is depreciation, which does not increase rapidly unless new buildings are constructed, and shows a downward trend over time. Therefore, if the labor costs continue to rise, the RIP will fall. With the exception of group 3, the RIP in the third period was higher than that in the first period in all of the years, and this was a factor in the deterioration of management. The year 2009, when significant investment resumed, was the year in which the government was replaced by the opposition. Thus, it is undeniable that the political pressure on the previous government in healthcare policy had an impact. In fact, the capital investment has been rapid since FY 2009 (Figure 4). This may have been an investment that made it impossible to maintain the replacement cycle of capital investment, which is clearly evident from the investment situation in the third period. When such unplanned investments are made, they include wasteful investments with a bias, such as that due to political pressure. Of course, the political influence is unavoidable when an independent administrative body operates under the approval and the direction of the administration. However, it may also be a turning point where the very strong political influence has made it difficult to maintain managerial independence. Despite the investment being lower than in the second period, the third period resulted in a negative FCF in cash flow, which did not generate the resources to repay the debt (Table 3).

Group 3 had stable management throughout the first two periods, and for this reason, it appeared that the group 3 hospitals were likely to be stable businesses, unless major capital investments were made (Figure 5c). However, in FY 2016, group 3 posted its only loss in 15 years. The reason for the deficit was the low growth rate of medical revenue, as shown by the decline in the RMP, and the increased growth rate of the personnel-related expenses of 7.2% and 2.8% between FY 2015 and FY 2016, respectively, which could not be covered (Table 3).

There was also an increase in the cost of materials in the acute care hospitals, particularly in groups 2 and 3 (Table 3). This may be due to the introduction of high-priced drugs in recent years. However, while the use of high-cost materials led to an increase in the medical revenue, it did not necessarily translate into medical profit. This was evident in the OPP of groups 2 and 3 in FY 2015 (Figure 5b,c). Furthermore, the large increase in the labor costs in 2015 was due to the fact that, although 50% of the labor insurance premiums are paid from the state treasury in the private sector, this was not applied to the NHO, and the subsequent policy changes increased the labor insurance premiums [18]. Although the growth rate of the personnel-related costs is presented for a single year, it is closely linked to the subsequent hospital management, and its impact over the medium- to long-term was seen for all of the RMP, RIP, and OPP indicators. This large increase in the labor costs in 2015 was not necessary for medical treatment, but purely due to political factors. These increases in labor costs, which have nothing to do with the medical practice and do not generate so-called medical income, naturally had a significant impact on the hospital management. This was a factor that caused most of the hospitals to suffer downward pressure on their management in the third phase.

Regarding the third period, during which the NHO had the worst business conditions, the revisions of the medical fees in FY 2014, FY 2016, and FY 2018 were +0.82%, +0.56%, and +0.63% for medical treatment, and −0.05%, −0.11%, and −0.09% for materials, respectively. These data at least indicate a positive revision for medical treatment. According to the 2021 white paper on small and medium enterprises in Japan, the BER (%) of small enterprises (with a capitalization of less than USD 0.09 million) is 90.9% (FY2018), which means that they are less resilient to crises [26]. In the NHO, only groups 1–4 in the first period had a BER that was below 0.9 (BER (%): 90%). In all of the other periods, the BER was above 0.9, and in the third period, it was almost 1 (Figure 7). The capital of the NHO was about USD 1.8 billion in 2020. In other words, the hospital management by independent administrative agencies in Japan is worse than the BER of a small company and is very vulnerable to a crisis.

The labor costs in the NHO have increased by an average of 3.3–3.4% in each period (Table 3). This is partly due to the increase in the number of staff, but also largely due to the pay structure of the organization. While the hospital director is empowered to increase or decrease the number of staff with the approval of the HQ, it is currently impossible for the NHO to make major changes to the staff salary structure, partly because it has taken over the national system. This analysis suggests that, under the current reimbursement system, even the natural increase in labor costs cannot be covered if strict public management continues after the transformation to an independent agency. In other words, if the public hospitals were legally (or in case implicitly) obliged to take on a public role after this transformation, they would be difficult to maintain and manage under the current reimbursement system. Of course, the long-term management of hospitals is possible if the official price of the medical care (reimbursement), which is revised every two years in Japan, is revised positively and can cover the higher labor costs in order to meet the political demands that are specially imposed on the public hospitals and are determined by a salary structure that is influenced by the national system. However, about 70% of the hospitals in Japan are private hospitals, which are free from policy objectives. The official prices are to be the same for all hospitals everywhere, and the official prices do not vary specifically by the public or the private sector. Therefore, a public pricing system is established with the main focus on the management of the private hospitals. This may be partly due to the historical setting of the medical fees in Japan by the Central Social Insurance Medical Council (CSIMC), which mainly operates through consultations between the private hospital owners and the insurers [1,27,28]. In fact, the representatives of the public hospitals are still not on the committee [29]. These hospitals were originally funded by public money and did not have to make a profit on the medical fees alone. However, this problem has arisen in recent years, as the public hospitals have had to generate income through the medical fees without changing their role or the cost structure after becoming independent administrative agencies.

The hospitals that are providing chronic care have increased their staffing levels in order to improve their earnings and to obtain higher levels of remuneration. In the reimbursement system in Japan, the basic fee is determined by the number of nurses that are assigned to a ward. Although this has improved the quality of care and the working environment, it has not led to a significant increase in the number of inpatients and, because of the nature of the patient population, it is unlikely to lead to a significant increase in remuneration. Thus, in order to improve management in the current situation, it is important for the acute hospitals to have an RMP above 0.25 so that the number of hospitals that are in surplus is greater than those that are in deficit. The BEP was also lower than 1 (Figure 8), which means that the RMP should be at least 0.25. For the chronic care hospitals (groups 4 to 6), the number of surplus hospitals is greater than the number of deficit hospitals when the RMP exceeds 0.21 and the BEP is also below 1 (Figure 9). This situation can be achieved by attracting more new patients or/and by reducing the labor costs in order to increase the RMP and by limiting the investment in order to reduce the RIP. In the NHO, functional restraint to the extent that is permitted by the legal system and a reduction in hospital beds have been pursued in the past. However, the OPP decreased to 0.001 in the third period. In addition, continuous negative FCF despite the reduction in the capital expenditure would create a shortage of facilities and financial inflexibility in a situation such as the COVID-19 pandemic that occurred in FY 2020.

If the independent administrative agencies are to become fully economically independent, the government should not impose special institutional restrictions in order to meet the political and administrative requirements. In order tobe more competitive in the market, it is essential to weaken the special political controls that are currently in operation and to create a freer structure that is aimed at a profit-making system with private management. However, this will make it more difficult to prepare for infectious diseases and disasters, which can put downward pressure on the business. This has also been pointed out as an explanation for the decline in the research power of national university medical departments, as a result of the change to an independent administrative agency. University hospitals now have to compete in the general healthcare market, which requires a concentration of management resources on the profitable areas (i.e., the medical treatment), rather than research [30]. In the event of a catastrophe or a pandemic, such as COVID-19, this could create a number of obstacles to a national or local government-led response. If the national government were to completely relinquish their ability to manage the situation, they would also lose the opportunity to have a free hand in the provision of healthcare, which is required by policy. In order to avoid such a situation, a higher public price for healthcare may be needed, including private hospitals, in order to allow for uncertainty in the normal course of events or a rapid provision of the necessary facilities in the event of a pandemic or a disaster. The same problem arises in local regions. Local government hospitals are required to respond to local health crises, similarly to the NHO; however, in order to decouple the local government hospitals from the local government finances, these hospitals are being converted into local independent administrative agencies. The results of this study suggest that the same problems might occur in municipal hospitals if they were to become independent administrative agencies, as is the case with the NHO.

Although the NHO provides a broad range of healthcare functions that are a microcosm of the Japanese healthcare delivery system, the study has the limitation that it cannot be adapted for the evaluation of efficiency in organizations that are more competitively managed when they are viewed on a hospital-by-hospital basis. The evaluation criteria are based on the personnel costs; however, if the safety and the functionality of the hospitals can be adjusted, comparisons can be made in terms of how well they are maintained and how much income they generate in relation to the personnel costs. On the other hand, as comparable treatment outcome data have not been taken in Japan, it is still difficult to conduct an efficiency assessment of costs based on outcomes.

## 5. Conclusions

The transformation of national hospitals and sanatoriums into independent administrative corporations was partly evaluated in this study as an improvement in profitability. However, in the third period of the study, there was an increasing tendency towards deficits. In particular, controlling the investment did not increase the profits and the FCF was negative for five years. This situation raises doubts about the long-term sustainability of independent management. Our results suggest that further organizational reforms are needed in order to sustain the country’s infrastructure in the long term. This is particularly important for health crisis management in areas such as infectious diseases, where continued investment is difficult. At the same time, it is important to evaluate and discuss the future of public hospitals, including the question of whether they should become independent administrative agencies.

## Figures and Tables

**Table 1:** Governance structures of health organizations; for more detailed information see Supplementary Material Table S1.

Classification by Type of Establishment	National	NHO	Public Hospital	Private
**Act on Basis for Establishment**	Law for Establishing Jurisdiction	Act on the National Hospital Organization, Independent Administrative Agency.	Local Public Enterprise Act/Articles of Incorporation	Medical Care Act.
**Establishers**	The competent minister	Chairman of the board of directors	Head of the local government/Business Manager	Hospital Administrator
**Appointing authority of the establisher**	The competent minister	Minister of Health, Labor and Welfare, National	Head of the local government	Hospital Administrators
**Status of the establisher**	Specialized National Public Servants	Non-government officer	Local government officer	Non-government officer
**Director Appointee**	-	The competent minister	-	Establishers
**Method of Election of Executive Board Members**	-	The competent minister	-	Board of directors/Establishers
**Management organization**	The ministry in charge	Board of directors	Hospital Organization	Board of directors/Establishers
**Operation Plans**	The ministry in charge	Board of directors	Hospital Organization/Local government that has established	Board of directors/Establishers
**Approval of operating plan**	The competent minister	The competent minister	Local government that has established	Board of directors/Establishers
**Approval of Management Report**	The competent minister/Council in charge	The competent minister/Council in charge	Local government that has established	Board of directors/Establishers
**Sponsor of a capital**	National government	National government	Local government that has established	Own private financial resources
**Budget Approval**	Congress	Board of directors	parliament	Board of directors/Establishers
**Financial Repor**	Congress	The competent minister	Ministry of Internal Affairs and Communications/Established local governments	Board of directors/Establishers
**Advisory board**	Council of Ministries and Agencies	Committee on the system of evaluating incorporated administrative agencies	Local Self-Governance Committee	Consultant firms, etc.
**Investment funds**	Special Accounts	FILP system/Own Assets	Municipal accounting	Own Assets/Bank
**Hospital Administrator (Hospital Director)**	A person who has been registered under Article 16-6, paragraph (1) of the Medical Practitioners’ Act as stipulated in Article 7, paragraph (1) of the Medical Care Act.			
**Hospital administrator’s appointee**	The competent minister	Chairman of the board of directors	Head of the local government	Chairman of the board of directors/Hospital Administrator
**Licensor for the establishment of hospitals**	Prefectural Governor			
**Limitations of opening**	Prefectural Governor			
**Authority to limit the number of hospital beds**	Prefectural Governor			
**Status of Employees**	Government officer	Private	Local government officer	Private
**Status under the Criminal Code**	Government officer	Public officer	Local government officer	Private
**Government’s right to command and control**	Has command and control authority.	The Minister of Health, Labor and Welfare may request the implementation of operations in the event of a disaster or public health crisis.	-	-
**Operational Supervisory Authority**	The competent minister	Prefectural Governor		
**Public audits on insurance treatment**	Regional Bureau of Health and Welfare (Ministry of Health, Labour and Welfare)			
**Surplus Profit**	Surpluses must in principle be paid into the national treasury	Surpluses after the end of the medium-term target period are managed by the agency for the next medium-term target period if they are approved by the competent minister, and if permission is not granted, the part of the surplus that has not been planned for use in the next plan must be returned to the national treasury.	Local government	Transfer of assets

**Table 2:** Final review of the medium-term management plan; for more detailed information see Supplementary Material Table S2.

Target item			Final review of medium-term		
Medium-term plan			1	2	3
I. Matters concerning the improvement of the quality of services and other operations provided to the public					
	Clinical Services				
		Providing Medical Care	A	A	B
		Providing Safe and Reliable Medical Care	A	A	
		High-Quality Medical Care	S	S	Reclassified.
		Contribution to National Health Care Policy	―	―	A
		Contribution to Local Medical Services	―	S	A
	Clinical Research Business		S	S	A
	Education and Training Business		A	S	B
II. Matters related to the efficiency of business management					
	Efficient Business Operation Structure		A	A	B
	Improvement of Efficiency of Business Operation, etc.		A	A	
	Effective Utilization of Medical Resources		S	S	
	Reduction of Expenses Related to Businesses Other Than Clinical Services, etc.		A	―	
	Promotion of Information Technology		A	―	
	Securing Revenue		―	A	
III. Matters Related to Improvement of Financial Status					
	Budget, Income and Expenditure Plan and Financial Plan		―	―	B
	Improvement of Management		S	A	
	Improvement of Fixed Liabilities Ratio		S	S	
IV. Other matters					
	Other Matters Concerning Business Operation as Provided for in the Ordinance of the Competent Ministry		A	A	B

The result of the rated score. “S”: much above target, “A”: target achieved, “B”: treated as achieved but some targets did not achieve. And there are even worse scores that the NHO has not received. “C”: failed to achieve targets overall, “D”: Improvements, including the discontinuation of operations, are required.

**Table 3:** Management indicators in the 15 years of the study.

	Group	2004	2005	2006	2007	2008	2009	2010	2011	2012	2013	2014	2015	2016	2017	2018	Average of 1st Period	Average of 2nd Period	Average of 3rd Period
Rate of increase in medical revenue	Group 1	-	−0.1%	0.3%	6.3%	2.6%	6.2%	9.1%	5.1%	2.7%	2.4%	0.8%	1.3%	3.3%	0.3%	4.3%	2.3%	5.1%	2.0%
	Group 2	-	3.3%	0.4%	6.7%	1.2%	5.9%	9.6%	4.3%	4.5%	2.5%	1.9%	5.1%	0.5%	3.6%	5.0%	2.9%	5.4%	3.2%
	Group 3	-	6.4%	2.3%	8.5%	2.7%	3.8%	7.8%	2.3%	3.7%	4.2%	1.8%	3.2%	0.9%	3.4%	3.8%	5.0%	4.3%	2.6%
	Group 4	-	5.9%	−2.4%	2.9%	3.3%	2.1%	2.9%	1.1%	0.4%	3.7%	2.2%	2.2%	−0.7%	0.6%	4.9%	2.4%	2.1%	1.8%
	Group 5	-	5.6%	2.7%	11.7%	7.0%	1.5%	2.4%	3.6%	−0.6%	2.3%	2.6%	−0.8%	−1.0%	3.5%	1.7%	6.7%	1.8%	1.2%
	Group 6	-	−0.8%	0.3%	3.6%	9.1%	2.6%	5.6%	2.5%	3.3%	8.3%	0.0%	3.1%	3.2%	4.1%	−3.1%	3.1%	4.5%	1.5%
	Total	-	3.4%	0.0%	5.6%	4.0%	3.7%	6.4%	2.9%	2.7%	4.0%	1.3%	2.2%	1.1%	2.1%	2.9%	3.3%	4.0%	1.9%
Rate of increase in personnel -related expenses	Group 1	-	1.7%	2.8%	6.9%	7.1%	4.2%	4.1%	5.6%	0.8%	2.9%	2.1%	5.9%	3.1%	0.7%	3.0%	4.6%	3.5%	2.9%
	Group 2	-	6.0%	1.5%	5.7%	3.1%	8.2%	4.4%	5.0%	4.0%	3.2%	4.0%	9.2%	2.2%	3.7%	4.9%	4.1%	4.9%	4.8%
	Group 3	-	8.9%	4.1%	8.2%	4.1%	5.3%	3.8%	4.4%	2.5%	5.9%	4.6%	7.2%	2.8%	2.0%	2.4%	6.3%	4.4%	3.8%
	Group 4	-	5.4%	−3.1%	1.7%	1.1%	1.6%	2.0%	1.8%	0.6%	2.6%	4.4%	7.1%	0.8%	0.9%	4.6%	1.3%	1.7%	3.6%
	Group 5	-	12.5%	2.6%	6.8%	−0.2%	1.4%	1.3%	1.1%	0.9%	2.7%	3.3%	5.4%	1.6%	1.6%	1.3%	5.4%	1.5%	2.6%
	Group 6	-	−1.0%	1.8%	3.1%	7.3%	3.9%	1.4%	3.4%	1.7%	7.5%	1.6%	8.4%	3.5%	2.8%	−3.2%	2.8%	3.6%	2.6%
	Total	-	4.6%	0.8%	4.5%	3.8%	4.1%	2.8%	3.5%	1.9%	4.1%	3.3%	7.0%	2.2%	1.7%	2.4%	3.4%	3.3%	3.3%
Rate of increase in material costs	Group 1	-	−3.1%	2.1%	4.2%	1.1%	7.5%	7.0%	4.2%	−1.7%	4.7%	−0.1%	1.0%	4.4%	−0.3%	4.0%	1.1%	4.3%	1.8%
	Group 2	-	3.9%	−0.1%	6.1%	0.0%	7.5%	4.4%	5.5%	2.0%	5.3%	3.1%	9.1%	2.1%	4.9%	5.5%	2.5%	4.9%	4.9%
	Group 3	-	6.9%	3.1%	7.1%	3.2%	6.2%	5.5%	4.0%	3.5%	7.0%	5.8%	7.5%	1.9%	2.9%	2.9%	5.1%	5.2%	4.2%
	Group 4	-	4.2%	−4.7%	4.0%	4.2%	5.1%	1.7%	1.0%	2.5%	6.1%	4.3%	5.4%	−1.5%	−0.5%	4.4%	1.9%	3.3%	2.4%
	Group 5	-	5.6%	−2.1%	4.9%	3.0%	0.8%	−0.5%	5.4%	4.5%	2.4%	−0.4%	5.7%	−3.5%	−3.0%	3.8%	2.9%	2.5%	0.5%
	Group 6	-	1.4%	0.8%	3.5%	11.2%	3.5%	4.0%	2.4%	2.7%	10.5%	1.9%	9.0%	7.8%	2.7%	−3.4%	4.2%	4.6%	3.6%
	Total	-	3.2%	0.3%	5.2%	3.6%	6.0%	4.4%	3.8%	2.0%	6.3%	2.8%	5.6%	2.8%	1.7%	2.8%	3.1%	4.5%	3.1%
Rate of increase in capital investme nt expenses	Group 1	-	−7.5%	−6.5%	1.0%	4.3%	26.7%	8.8%	4.4%	3.8%	13.2%	3.7%	−7.2%	0.8%	0.2%	−0.8%	−2.2%	11.4%	−0.6%
	Group 2	-	11.4%	0.3%	−1.0%	4.5%	44.3%	8.2%	1.6%	6.1%	3.9%	3.8%	−8.0%	−1.3%	1.7%	7.2%	3.8%	12.8%	0.7%
	Group 3	-	2.6%	−11.6%	−0.7%	1.2%	32.8%	8.7%	0.8%	5.9%	11.9%	−0.1%	−11.7%	−3.4%	−3.7%	−1.1%	−2.1%	12.0%	−4.0%
	Group 4	-	13.2%	−14.0%	−2.5%	3.1%	24.4%	6.3%	1.6%	4.2%	12.2%	4.0%	−7.8%	−2.1%	4.3%	4.9%	0.0%	9.7%	0.7%
	Group 5	-	18.0%	−7.5%	−4.4%	1.6%	27.7%	7.4%	−10.9%	11.0%	8.0%	0.7%	−6.5%	−5.9%	0.7%	3.5%	1.9%	8.6%	−1.5%
	Group 6	-	1.3%	−10.4%	−1.4%	7.6%	27.9%	2.6%	2.3%	4.3%	19.6%	−3.6%	−8.3%	6.2%	6.2%	−3.7%	−0.7%	11.3%	−0.7%
	Total	-	6.0%	−8.9%	−1.4%	4.1%	31.1%	6.6%	1.3%	5.2%	11.5%	1.3%	−8.8%	−0.3%	2.0%	2.0%	0.0%	11.1%	−0.8%
FCF (US$1 million)	Group 1	−34.4	11.2	1.4	6.3	−6.8	−12.5	−26.4	−24.5	−7.7	−8.0	−51.6	−39.9	3.3	−4.9	36.0	−4.5	−15.8	−11.4
	Group 2	36.7	−22.8	25.6	72.4	−103.5	−142.2	52.5	130.2	131.8	112.3	38.6	−31.1	18.7	2.2	−151.1	1.7	56.9	−24.5
	Group 3	45.7	113.4	139.3	141.5	123.9	78.7	145.4	96.0	97.3	119.0	81.2	89.7	−19.9	71.7	22.0	112.8	107.3	48.9
	Group 4	−23.6	24.2	75.2	71.8	74.0	75.6	73.3	58.9	31.0	−27.8	−63.5	−18.7	33.0	−34.4	−61.4	44.3	42.2	−29.0
	Group 5	−14.4	−1.5	−7.8	9.6	21.4	23.8	17.2	27.4	4.1	3.6	−21.0	−51.5	−2.1	−4.0	−3.1	1.5	15.2	−16.3
	Group 6	4.4	42.9	52.7	53.1	51.3	37.8	39.6	56.8	71.7	−47.4	−49.0	−51.6	−77.9	−118.2	−6.7	40.9	31.7	−60.7
	Total	14.4	167.4	286.5	354.6	160.2	61.3	301.7	344.7	328.3	151.8	−65.3	−103.0	−44.9	−87.6	−164.3	196.6	237.6	−93.0
Number of Beds	Group 1	4854	4708	4558	4352	4014	3922	3899	3884	3929	3693	3793	4187	4187	4274	4222	4408	3865	4133
	Group 2	9454	9751	9858	9767	9784	9631	9728	9775	9735	9744	9725	9295	9271	8851	8711	9790	9723	9171
	Group 3	8420	8412	8384	8357	8357	8369	8350	8324	8299	8164	8103	8051	8019	7984	7832	8378	8301	7998
	Group 4	13,934	13,881	13,986	13,819	13,601	13,529	13,388	13,331	13,235	13,218	13,570	14,747	14,851	14,761	14,846	13,822	13,340	14,555
	Group 5	4963	4816	4838	4574	4555	4554	4458	4459	4386	4322	4306	4312	4362	4291	4284	4696	4436	4311
	Group 6	13,263	13,727	13,450	13,164	12,724	12,493	12,639	12,501	12,366	12,471	11,968	10,641	10,648	10,975	10,795	13,266	12,494	11,005
	Total	54,888	55,295	55,074	54,033	53,035	52,498	52,462	52,274	51,950	51,612	51,465	51,233	51,338	51,136	50,690	54,359	52,159	51,172

## Data Availability

All data in the study will be made available upon reasonable request to the corresponding author.

## Supplementary Materials

The following supporting information can be downloaded at: https://www.mdpi.com/article/10.3390/healthcare10102084/s1, Table S1: Governance structures of health organizations (detailed); Table S2: Final review of the medium-term management plan (detailed).
