# Supplementary material for: Hospital Management and Public Health Role of National Hospitals after Transformation into Independent Administrative Agencies
Source: Healthcare (Basel). 2022 Oct 19;10(10):2084. doi: 10.3390/healthcare10102084 (PMC9602203; doi:10.3390/healthcare10102084)
Supplement: Supplementary file 1 [file healthcare-10-02084-s001.zip › healthcare-1944592-supplementary.pdf]

Table S1. Governance structures of health organizations(detailed).

Classification by type of establishment	National Hospital	Independent Administrative Agency (National)	Public Hospital	Local Independent Administrative Agency	Private Hospital
Example	National Defense Medical College Hospital, National Sanatorium for Hansen's Disease	NHO, National University Hospitals, etc.	Local government hospital	Hospitals operated under the Local Public Enterprise Act	Medical corporations, clinics, etc.
Act on Basis for Establishment	Law for Establishing Jurisdiction	Act on General Rules for Incorporated Administrative Agencies. / Act on the National Hospital Organization, Independent Administrative Agency. / Act on the National University Corporation.	Local Public Enterprise Act / Articles of Incorporation	Local Public Enterprise Act / Articles of Incorporation	Medical Care Act.
Establishers	The competent minister	Chairman of the board of directors	Head of the local government / Business Manager	Chairman of the board of directors	Chairman of the board of directors / Hospital Administrator
Appointing authority of the establisher		The competent minister / Ex. NHO: Minister of Health, Labor and Welfare, National University Hospitals: Ministry of Education, Culture, Sports, Science and Technology.	Head of the local government	head of the local government	Chairman of the board of directors / Hospital Administrators
Status of the establisher		Non-government officer	Local government officer	Non-government officer	Non-government officer
Director Appointee	The competent minister	The competent minister	—	Head of the local government	Establishers
Method of Election of Executive Board Members	Specialized National Public Servants	The competent minister / Open recruitment	—	Head of the local government / Open recruitment	Board of directors / Establishers

Classification by type of establishment	National　Hospital	Independent Administrative Agency (National)	Public Hospital	Local Independent Administrative Agency	Private Hospital
Management organization	The ministry in charge	Board of directors	Hospital Organization	Board of directors	Board of directors / Establishers
Operation Plans	Hospital Organization / The ministry in charge	Board of directors	Hospital Organization / Local government that has established	Board of directors	Board of directors / Establishers
Approval of operating plan	The competent minister / Council in charge	The competent minister / Council in charge	Local government that has established	Parliament	Board of directors / Establishers
Approval of Management Report	The competent minister / Council in charge	The competent minister / Council in charge	Local government that has established	parliament	Board of directors / Establishers
Sponsor of a capital	National government	National government	Local government that has established	Local government that has established	Own private financial resources
Budget Approval	Congress	Board of directors	parliament	Board of directors	Board of directors / Establishers
Financial Repor	Congress	The competent minister	Ministry of Internal Affairs and Communications / Established local governments	Ministry of Internal Affairs and Communications / Established local governments	Board of directors / Establishers
Advisory board	Council of Ministries and Agencies	A Committee on the System of Evaluating Incorporated Administrative Agencies are set up within the Ministry of Internal Affairs and Communications. And this committee makes recommendations to the competent minister.	Local Self-Governance Committee	Local Self-Governance Committee	Consultant firms, etc.
Investment funds	Special Accounts	FILP system / Own Assets	Municipal accounting	FILP system / local government / Bank	Own Assets / Bank

Classification by type of establishment	National Hospital	Independent Administrative Agency (National)	Public Hospital	Local Independent Administrative Agency	Private Hospital
Hospital Administrator (Hospital Director)	A person who has been registered under Article 16-6, paragraph (1) of the Medical Practitioners' Act as stipulated in Article 7, paragraph (1) of the Medical Care Act.				
Hospital administrator's appointee	The competent minister	Chairman of the board of directors	Head of the local government	Chairman of the board of directors	Chairman of the board of directors / Hospital Administrator
Licensor for the establishment of hospitals	Prefectural Governor:<br>Licensing of buildings (additions and renovations, changes and additions to medical equipment, use of rooms, changes to room names), number of beds and establishment (ICU, CCU, etc.), and licensing of medical departments.<br>Permission for buildings (additions and renovations, changes and additions to equipment, use of individual rooms, changes to room names), and permission for hospital beds (number of beds, etc.).<br>Permission for a medical speciality.				
Limitations of opening	Prefectural Governor: Must permit if the building structure, equipment and staffing standards are met.				
Authority to limit the number of hospital beds	Prefectural Governor: In principle, within the scope of the regional medical plan drafted in accordance with the Medical Care Act.				
Notification related to medical reimbursement under health insurance laws	Regional Health and Welfare Bureau (Ministry of Health, Labour and Welfare)				

Classification by type of establishment	National　Hospital	Independent Administrative Agency (National)	Public Hospital	Local Independent Administrative Agency	Private Hospital
Status of Employees	Government officer	Private	Local government officer	Private	Private
Status under the Criminal Code	Government officer	Public officer	Local government officer	Private	Private
Government's right to command and control	Has command and control authority.	Depends on the Act.　/ Ex. The Minister of Health, Labor and Welfare may request the implementation of operations in the event of a disaster or public health crisis as defined in Article 21.1 of the Act on the National Hospital Organization, Independent Administrative Agency.	—	—	—
Operational Supervisory Authority	The competent minister	Prefectural Governor: Comply with the provisions of Section 3 of the Medical Care Act (staffing, sanitation, compliance with laws and regulations)			
Public audits on insurance treatment	Regional Bureau of Health and Welfare (Ministry of Health, Labour and Welfare)				
Surplus Profit	Surpluses must in principle be paid into the national treasury	Surpluses after the end of the medium-term target period are managed by the agency for the next medium-term target period if they are approved by the competent minister, and if permission is not granted, the part of the surplus that has not been planned for use in the next plan must be returned to the national treasury.	Local government	Transfer of assets	Transfer of assets

Table S2. Final review of the medium-term management plan(detailed).

Target item			Final review of medium-term					
Medium-term plan			1st		2nd		3rd	
I . Matters concerning the improvement of the quality of services and other operations provided to the public								
Clinical Services								
Providing Medical Care								
Patient Satisfaction Survey	Increase in average satisfaction points	Inpatients	4,310pt→4,508pt		4,508pt→4,545pt		4,549pt→4,554pt	
		Outpatient	3,920pt→4,097pt		4,097pt→4,122pt		4,141pt→4,148pt	
Clinical-Path	The number of completed clinical-path	Increase in variety	5.2k→7.5K		―		―	
	The number of cases conducted	Increase in the number of cases	126.8K→243.7K		243.7K→288.4K		288.4K→319.7K	
Second Opinion	The number of hospitals Implementing the Program	Increase in the number of hospitals implementing the program	45→129		129→143		―	
	The number of cases conducted	Increase in the number of cases	1,228→2,928		2,928→3,480		―	
Issue of Statements of Account	The number of hospitals with Issuing system in place	Inpatients	59→70		―		―	
		Outpatient	52→66		―		―	
	Issuance of a statement clearly indicating individual calculation items	Increase in the number of hospitals implementing issuance	―		8→143		―	
Assignment of Medical Social Worker	The number of deployments	The number of hospitals	55→113		113→137		137→137	
		The number of deployments	71→229	A	229→412	A	412→537	

Target item			Final review of medium-term		
Medium-term plan			1st	2nd	3rd
Committee for Quality Improvement of Health Care	The number of hospitals	Installation in all facilities	—	—	5→141
Ordinary Income	Ordinary income	Return to profitability in recurring income	—	—	US$355.5M→ US$126.6M→US$46.6M →US$97.1M→
The number of Employees	The number of employees	The number of employees	—	—	59.3K→60.1K→ 61.1K→ US$116.0M
Providing Safe and Reliable Medical Care					
Provide a private room where patients can consult.	The number of hospitals that have converted to private rooms		105→126	126→132	—
Ethics Review Board	Installation and Implementation	The number of hospitals with	91→all	—	—
		The number of times held	382→628	628→893	Reclassified.
		The number of cases reviewed	1.2K→2.4K	2.4K→4.7K	Reclassified.
Clinical Trials Review Committee	Installation and Implementation	The number of hospitals with	129→all	—	—
		The number of times held	—→1.1K	1.1K→1.0K	Reclassified.
		The number of cases reviewed	—→14.0K	14.0K→14.8K	Reclassified.

B

Target item						Final review of medium-term					
Medium-term plan						1st		2nd		3rd	
			Report of Medical Accident	Thorough reporting of incidents	The number of reported cases	112→728		—		201→242	
			Standardization of medical equipment	Limitation of the ventilator to designated models	Increase in percentage of designated models	35.4%→54.2%		54.2%→89.4%		90.3%→94.5%	
			Hospital infection control	Installation of ICT	Increase in the number of hospitals	97→137		—		—	
				The number of certified infection control nurses	Increase in the number of nurses	33→87	A	87→155	A	155→214	
				Training on hospital infection control	Increase in the number of training	—		618→1,012		1,012→1,390	
			Medical Safety	The number of new nurses who attended medical safety training	Increase in the number of trainees	—→3,9K		3.9K→7.0K		—	
				Conducting medical safety workshops	Increase in the number of workshops	—→22		22→14		14→18	
					The number of participants	—		927→486		486→733	

Target item			Final review of medium-term		
Medium-term plan			1st	2nd	3rd
Specialized and Certified Nurses	The number of specialized and certified nurses	The number of hospitals with	(39→81)	(86→119)	119→134
		Increase in the number of staff assigned	(74→285)	(258→686)	686→1,103
Accuracy of Laboratory Data	Clinical Laboratory Accuracy Management Survey" conducted by the Japan Medical Association	The average value of NHO	—	—	98.9→99.0→98.8→98.5 →98.3(max 100)
		National average.	—	—	97.3→97.2→97.0→97.1 →97.0(max 100)
Improvement of emergency medical care	The number of emergency patients accepted	The number of acceptances	584.1K→618.8K→634.5K →627.7K→564.8K	Reclassified.	—
	The number of pediatric emergency patients accepted	The number of acceptances	165.1K→169.0K→174.6K →160.3K→139.8K	Reclassified.	—
	Emergency and critical care center	Increase in the number of installations	14→17	Reclassified.	—
	24-hour pediatric emergency system	Increase in the number of well-structured hospitals	11→17	Reclassified.	—

Target item				Final review of medium-term					
Medium-term plan				1st		2nd		3rd	
High-Quality Medical Care									
	Medical Related Workshops	The number of participants	Increase in the number of participants	1.8K→2.0K		1,7K→1.8K		—	
	Assignment of medical care workers	Hospitals where placements were made	Increase in the number of facilities placed	—→49		49→68		Reclassified.	
		The number of people assigned	Increase in the number of staff assigned	—→563		563→1,154		Reclassified.	
	Day-care facilities for severely mentally and physically handicapped persons	Increase in the number of hospitals installed	Increase in the number of hospitals installed	21→28		—		—	
	Medical Care for Tuberculosis	Percentage of domestic medical treatment	Inpatients	45%		—		—	
		Average days in the hospital	the shorter average length of hospital stay	77.6→73.2	S	—	S	—	—

Target item						Final review of medium-term		
Medium-term plan						1st	2nd	3rd
			Rate of referrals	Rate of referrals	Increase of more than 5 per cent	40.5→53.9%	53.9%→64.7%	Reclassified.
				Reverse Referral Rate	Increase of more than 5 per cent	28.7%→42.7%	42.7%→52.6%	Reclassified.
			Medical treatment for cancer	Prefectural cancer treatment center hospital	Increase in the number of center hospitals	0→2	Reclassified.	—
				Regional cancer treatment base hospital	Increase in the number of base hospitals	11→31	Reclassified.	—
			Regional Medical Support Hospital	Efforts to Designate	Increase in the number of designated hospitals	4→33	Reclassified.	—
		Contribution to National Health Care Policy						
			Generic drug	Utilization rate based on volume	Utilization rate	—	(16.4%→33.5%)	66.4%→72.7%→78.7%→83.5%→86.2%
				Utilization rate based on the value	Projected value	—	—	60%→60%→70%→70%→70%

Target item						Final review of medium-term		
Medium-term plan						1st	2nd	3rd
			DMAT Training	Training Implementation	Increase in the number of facilities	(7→119)	—	50→232
					Increase in participation	(35→475)	—	505→919
				Supervision DMAT Training	Securing Participants	—	—	105→120
				Skills maintenance training for DMAT members	The number of facilities	—	—	883→1,195
					Participants	—	—	3,008→3,341
			Disaster Training	Disaster Medical Staff Training 1	Participants	—	—	79→80→82→68→78
				Disaster Medical Staff Training 2	Participants	—	—	—→29→39→31→32
				First Response Medical Team Training	Participants	—	—	71→63→65→72→43
				Disaster Medical Care Training	Participants	—	—	58→71→91→15→71

Target item						Final review of medium-term					
Medium-term plan						1st		2nd		3rd	
			Assignment of medical assistance personnel	Staffing Hospitals	Increase in the number of staffed facilities	(—→49)		(49→68)		68→73	
				The number of Staff	Increase in the number of staffing	(—→563)		(563→1,154)		1,154→1,378	
			Secretariat of the Liaison Council for Medical Care of Intractable Diseases	The number of hospitals with administrative offices	Increase in the number of facilities with administrative offices	—		—		—→ 6	
			Support Center for Intractable Disease Consultation	The number of hospitals with centers	Increase in the number of facilities with administrative offices	—	—	—	—	4→ 7	A
			Implementation of therapeutic research projects for specific diseases	The number of patients admitted to hospital	The number of acceptances	—		—		1.3M→1.4M→1.4M→ 1.5M→1.5M	
			Project for Research and Treatment of Specific Chronic Childhood Diseases	The number of patients admitted to hospital	The number of acceptances	—		—		76.7K→76.1K→72.4K→ 73.9K→77.0K	

Target item						Final review of medium-term		
Medium-term plan						1st	2nd	3rd
			Psychiatry	Drug dependence	Total number of inpatients	—	—	14.2K→14.1K→12.7K→12.5K→8.9K
				Alcohol dependency	Total number of inpatients	—	—	89.4K→82.2K→87.9K→86.1K→86.3K
				Psychiatric Emergencies	Total number of inpatients	—	—	6.9K→7.6K→9.0K→3.3K→3.2K
				Dementia Medical Center	The number of designated hospitals	—	—	8→9→12→13→13→13
			Medical Observation Law Ward	The number of admissions per day	Acceptance of hospitalization	—	(346.3→408.3)	404.9→384.3→359.0→375.4→371.4
			Medical Care for Tuberculosis	Total number of inpatients	Acceptance Implementation	—	(564.7K→351.8K)	325.3K→298.7K→275.0K→254.9K→231.2K
				of which multidrug-resistant tuberculosis	Acceptance Implementation	—	—	11.5K→9.1K→6.6K→7.6K→7.2K→5.4K
				The number of beds	The number of beds	—	—	2.2K→2.0K→2.0K→1.9K→1.6K
				Bed utilization rate	Improvement of bed utilization	—	(58.7%→53.4%)	54.0%→52.8%→52.0%→48.7%→49.1%
				DOTS implementation rate	Promotion of implementation	—	—	99.5%→98.2%→98.5%→98.3%→97.8%

Target item				Final review of medium-term		
Medium-term plan				1st	2nd	3rd
Contribution to Local Medical Services						
Regional Critical Paths	Regional Critical Paths	Increase of more than 5 per cent		—	2.1K→3.2K	6.6K→8.8K
Regional Medical Support Hospital	Efforts to Designate	Increase in the number of hospitals		(4→33)	33→57	57→59
Rate of referrals	Rate of referrals	Increase of more than 5 per cent		(40.5→53.9%)	(53.9%→64.7%)	64.7%→78.1%
	Reverse Referral Rate	Increase of more than 5 per cent		(28.7%→42.7%)	(42.7%→52.6%)	52.6%→64.1%
Medical treatment for cancer	Prefectural cancer treatment center hospital	Increase in the number of center hospitals		(0→2)	2→3	3→3
	Regional cancer treatment base hospital	Increase in the number of base hospitals		(11→31)	31→35	35→33
	Regional Cancer Hospital	Increase in the number of hospitals		—	—	0→ 1
	Cancer Genome Medical Cooperation Hospital	Increase in the number of hospitals		—	—	—→10

Target item							Final review of medium-term		
Medium-term plan							1st	2nd	3rd
			Response to the Great East Japan Earthquake	Medical Support	Disaster Medical Assistance Team		—	340 Team	—
				The number of emergency patients accepted	Implementation of patient acceptance		(584.1K→618.8K→634.5K→627.7K→564.8K)	593.0K→563.7K→560.1K→546.0K→531.3K	533K→537.4K→548.6K→541.2K→536.7K
				The number of pediatric emergency patients accepted	Implementation of patient acceptance		(165.1K→169.0K→174.6K→160.3K→139.8K)	161.4K→138.4K→127.8K→130.0K→117.2K	112.9K→114.1K→110.7K→106.4K→101.1K
				The number of hospitalizations after emergency visits	Implementation of patient acceptance		—	153.4K→159.4K→163.8K→161.4K→161.4K	172.1K→176.8K→181.6K→183.3K→187.1K
				The number of hospitalizations after emergency visits for children	Implementation of patient acceptance		—	24.3K→22.8K→22.0K→20.1K→19.0K	19.4K→20.2K→21.7K→19.4K→20.8K

Target item						Final review of medium-term					
Medium-term plan						1st		2nd		3rd	
			Improvement of emergency medical care	The number of ambulances accepted	Implementation of patient acceptance	—	—	134.2K→146.1K→150.8K→154.6K→159.1K	S	165.6K→176.8K→180.4K→186.8K→194.9K	A
				The number of pediatric emergency patients transported by ambulance	Implementation of patient acceptance	—		10.8K→11.0K→11.0K→11.0K→11.5K		12.0K→12.4K→13.5K→14.3K→15.0K	
				The number of hospitalizations after ambulance visits	Implementation of patient acceptance	—		76.3K→82.4K→84.5K→85.9K→87.8K		92.1K→95.0K→100.1K→104.6K→106.8K	
				The number of children hospitalized after being transported by ambulance	Implementation of patient acceptance	—		—		4.2K→4.6K→4.7K→4.9K→4.9K	
				Emergency and critical care center	Increase in the number of hospitals with administrative offices	(14→17)		17→18		18→20	
				24-hour pediatric emergency system	Increase in the number of hospitals with systems in place	(11→17)		17→15		15→17	

Target item						Final review of medium-term					
Medium-term plan						1st		2nd		3rd	
			Medical Center for Intractable Diseases	The number of Hospitals	Establishment	—		—		28→26	
			Cooperative Medical Hospitals for Intractable Diseases	The number of Hospitals	Establishment	—		—		61→55	
			Medical Observation Law Ward	Hospitals with established wards	Increase in the number of hospitals	—		12→14		—	
				The number of beds	Securing the number of beds	—		359→421		—	
				The number of admissions per day	Acceptance of hospitalization	—		346.3→408.3		Reclassified.	
			Medical Care for Tuberculosis	Total number of inpatients	Implementation of patient acceptance	—		564.7K→351.8K		Reclassified.	
				Bed utilization rate	Improvement of bed utilization rate	—		58.7%→53.4%		Reclassified.	
			Generic drug	Utilization rate based on volume	Increase in utilization rate	—		16.4%→33.5%		Reclassified.	
				Utilization rate in value terms	Increase in utilization rate	—		8.3%→10.0%		—	

Target item				Final review of medium-term		
Medium-term plan				1st	2nd	3rd
Clinical Research Business						
	Promotion of Large-Scale Clinical Research	The number of Tasks	End	9	—	7
			Continue	5	30	22
	Efforts against the New Influenza Pandemic	Goals to be achieved in vaccine clinical trials	370 cases	Achieved in 1 month	—	—
		The number of clinical studies		—	5	—
	Acquisition of policy-designated research at the Ministry of Health, Labor and Welfare	The number of Tasks		11	—	—
	Academic Activities	English original papers	The number of papers (more than 5% increase)	—	1.3K→1.9K	2.1K→2.3K→2.4K→2.5K →2.6K
		The original paper in Japanese	The number of Papers	—	1.6K→1.7K	1.7K→1.8K→1.7K→1.7K →1.5K
		Presentations at International Conferences	The number of presentations	—	0.6K→1.2K	1.2K→1.1K→1.4K→1.5K →1.4K
		Presentations at Domestic Conferences	The number of presentations	—	11.1K→19.0K	21.0K→21.0K→20.4K→ 19.6K→18.7K
	Obtaining research funding	Obtaining research funding	Increase in research expenses	(US$16.3M→US$24.5M)	(US$24.5M→ US$24.7M)	US$29.5M→US$30.5M →US$30.6M→ US$30.5M→US$26.6M

Target item					Final review of medium-term					
Medium-term plan					1st		2nd		3rd	
		Clinical Trials	The number of hospitals with clinical trial coordinators	Increase in the number of facilities with administrative offices	27→64		64→70		70	
			The number of clinical trial coordinators	Increase in the number of staff assigned	54→153	S	153→209	S	209→245	A
			The number of Clinical Trials	Increase in the number of cases	2.8K→4.3K		4.3K→4.2K		4.8K→3.9K	
			Sales from Clinical Trials	Increase in sales amount	US$32.6M→US$43.9M		US$43.9M→US$41.6M		US$45.5M→US$42.3M	
		Restructuring of Clinical Research Organization	Clinical Research Center	The number of Centers	—		10→12		12→10	
			Clinical Research Division	The number of Clinical Research Divisions	—		60→71		71→77	
			Clinical Research Division (in-house advocates)	The number of clinical Research Division (in-house advocates)	—		43→47		47→45	

Target item					Final review of medium-term					
Medium-term plan					1st		2nd		3rd	
		Ethics Review Board	The number of times held, number of examinations	The number of times held	(382→628)		(628→893)		893→982	
				The number of examinations	(1.2K→2.4K)		(2.4K→4.7K)		4.7K→7.2K	
		Clinical Trials Review Committee	The number of times held, number of examinations	The number of times held	(—→1.1K)		(1.1K→1.0K)		1.0K→1.0K	
				The number of examinations	(—→14.0K)		(14.0K→14.8K)		14.8K→21.1K	
		COI Review Committee	The number of times held, number of examinations	The number of times held	—		—		343→423	
				The number of examinations	—		—		2.2K→3.2K	

Target item				Final review of medium-term					
Medium-term plan				1st		2nd		3rd	
Education and Training Business									
	Physician Training	Junior Residents.	Increase in the number of accepted	559→713		713→725		725→922	
		Senior Residents	Increase in the number of accepted	799→816		802→845		845→909	
		Training workshops for clinical internship supervisors	Increase in the number of times	5→5		—		—	
			Increase in participation	177→190		—		146→188→177→177→127	
	registered nurse	Assignment of a full-time Education Division Chief	Increase in the number of facilities	0→45		45→103		103→120	
		The number of specialized/certified nurses	The number of Hospitals Assigned	39→81		86→119		Reclassified.	
			Increase in the number of staff assigned	74→285		258→686		Reclassified.	
		Training of Practice Leaders	The number of facilities where the event was held	1 →6		6→6		—	
			The number of Lecturers	52→261	A	261→245	S	245→222	B

Target item				Final review of medium-term		
Medium-term plan				1st	2nd	3rd
		Scholarship Loans	The number of people loaned	20→131	131→1,876	Reclassified.
			The number of loan recipients employed	14→53	53→761	Reclassified.
		The pass rate of national examinations	OIST Specialty Schools	98%	98.5%	98.4%
			National Average	92.7%	94.9%	95.1%
	Midwife	The pass rate of national examinations	Professional school in OIST	—	98.2%	99.8%
			National Average	—	94.8%	98.4%
	DMAT Training	The number of Hospitals Conducting Training	Increase of hospitals	7→119	—	Reclassified.
			Increase in participation	35→475	—	Reclassified.
	Medical Training in the Community	The number of community health care training	Increase of 15% or more	—	2.2K→3.5K	4.7K→5.2K
	Training to improve the quality and standardization of	Participants in the training	Participant	—	—	103→76→75→57→81

Target item				Final review of medium-term		
Medium-term plan				1st	2nd	3rd
Ⅱ. Matters related to the efficiency of business management						
Efficient Business Operation Structure						
	Training to improve hospital management strategy capacity	Training 1. to improve hospital management strategy capacity	Participant	—	—	141→137→116→201→194
		Training 2. to improve hospital management strategy capacity	Participant	—	—	139→164→127→232→225
	Compliance	Voluntary compliance inspections	All hospitals	—	—	137→All hospitals
	Implementation of management guidance	The number of Implementation of management guidance	The number of hospitals	13→28→23	—	—
	Reduction of staff numbers	Reduction of technical staff	No supplement	1207	875	Reclassified.
	Outsourcing	Clinical Laboratory	The number of hospitals that have implemented	3→8	7	—
		Food preparation	The number of hospitals that have implemented	5→8	17	—

Target item					Final review of medium-term					
Medium-term plan					1st		2nd		3rd	
		Introduction of staff performance evaluation	All hospitals	All hospitals	all hospitals	A	all hospitals	A	all hospitals	
			Bonuses linked to management performance	All hospitals	all hospitals		all hospitals		all hospitals	
		End-of-year bonus	Implementation of end-of-year bonuses at hospitals in the black	The number of hospitals	42→30→28→62→56		Reclassified.			
		Reduction of training schools	Nurses	Reduction of training schools	68→42		—		—	
			Midwife	Reduction of training schools	5→5		—		—	
			Rehabilitation	Reduction of training schools	6→1		—		—	
			Vision Trainer	Reduction of training schools	1→1		—		—	
		Consolidation of hospitals	Hospital consolidation (up to 10 locations)	The number of integrated hospitals	8		2			
			Abolished	The number of hospitals eliminated	1		—		—	

Target item				Final review of medium-term		
Medium-term plan				1st	2nd	3rd
Improvement of Efficiency of Business Operation, etc.						
	Achievement of profitability	The number of hospitals in the black	Increase in the number of hospitals	—	111→122→117→123→113	—
		The number of deficit hospitals	Decrease in the number of hospitals	—	31→20→25→19→29	—
	Year-end bonus	Implementation of end-of-year bonuses at hospitals in the black	The number of hospitals	(42→30→28→62→56)	57→117→102→98→92	—
	Efficiency of hospital beds	General hospital beds	The number of beds reduced	—	861	—
		Beds for tuberculosis	The number of beds reduced	—	665	—
		Beds for Mental Illness	The number of beds reduced	—	276	—
	Training in Medical Affairs	Ensure the number of participants	Ensure the number of participants	—	127→134	121→92

Target item					Final review of medium-term					
Medium-term plan					1st		2nd		3rd	
		Training for proper billing of medical fees	Ensure the number of participants	Ensure the number of participants	—		—		121→125	
		Hospital Management Training Program	Ensure the number of participants	Ensure the number of participants	—		280→138		—	
		Implementation of joint bidding	The number of participating hospitals	The number of participating hospitals	101→all hospitals		—		—	
			Types of Drugs	Increase of items	5.9K→13.6K		—		—	
		Inventory assets	Assets (Pharmaceuticals)	Reduction	US$31.6M→US$29.2M	A	US$29.2M→US$40.3M	A	—	
			Inventory turnover days (Pharmaceuticals)	Reduction	14.2 days→11.9 days		11.9 days→13.0 days		—	
			Assets (medical supplies)	Reduction	US$28.2M→US$17.4M		US$17.4M→US$14.9M		—	
			Inventory turnover (medical supplies)	Reduction	20.3 days→11.2 days		11.2 days→8.2 days		—	B

Target item					Final review of medium-term		
Medium-term plan					1st	2nd	3rd
		Supply Processing Distribution; SPD	The number of hospitals introduced	Increase in the number of hospitals	—	77→88	—
		Material Cost Ratio	Material Rates	Maintain or control	—	24.0%→24.3%	—
		The number of surgeries	No reduction in the number of surgeries while keeping inventories under control	Maintaining or increasing	158.9K→174.3K	—	—
		Personnel rate + Commissioning rate	Control Personnel rate + Commissioning rate	Plan	59.1%→58.6%→58.8%→58.2%→58.3%	—	
				Actual value	58.8%→57.8%→58.1%→57.4%→57.0%	57.4%→55.3%→55.8%→55.3%→55.4%	56.3%→58.7%→59.4%→59.2%→58.8%
		Reduction of administrative expenses	Reduce administrative costs	Decrease in amount	US$33.4M→US$31.0M	—	—
			General Management Fee	5% reduction	—	US$6.8M→US$5.2M	US$4.9M→US$6.0M
		Reduction of labour costs	Reduce labour costs		US$2.8B→US$2.9B	US$3.1B(FY2011)	—

Target item				Final review of medium-term				
Medium-term plan				1st		2nd		3rd
Effective Utilization of Medical Resources					S		S	
The number of medical devices in operation	CT	Increase in the number of cases	759.1K→944.9K		944.9K→1.1M		—	
	MRI	Increase in the number of cases	280.6K→381.6K		381.6K→434.9K		—	
	Gamma cameras	Increase in the number of cases	102.5K→82.4		—		—	
	Per CT unit	Increase in the number of cases	4.1K→5.3K		5.3K→5.9K		—	
	Per MRI unit	Increase in the number of cases	2.2K→2.8K		2.8K→2.9K		—	
	Per Gamma camera unit	Increase in the number of cases	1.0K→2.8K		—		—	
Joint use with other institutions	CT	Increase in the number of cases	13.5K→28.5K		28.5K→36.0K		—	
	MRI	Increase in the number of cases	11.4K→27.6K		27.6K→36.7K		—	
	Gamma camera	Increase in the number of cases	3.4K→2.9K		—		—	
Investment in medical equipment	Investment Limit US$454.5M	Actual amount	US$637.3M		Reclassified.		—	
Investment	Investment Limit US$1.3B	Actual amount	US$1.2B		Reclassified.		—	
Reduction of Expenses Related to Businesses Other Than Clinical Services, etc.				A		—		
Obtaining research funding	Obtaining research funding	Increase in research expenses	US$16.3M→US$24.5M		Reclassified.		—	

Target item					Final review of medium-term					
Medium-term plan					1st		2nd		3rd	
	Promotion of Information Technology					A		—		
		Implementation of financial accounting system	Introduction of the system	Introduction of the system	Already in place		—		—	
		Introduction of electronic medical records	Introduction of electronic medical records	The number of hospitals with EMRs	—		—		91→102→104→110→114	
	Securing Revenue					—		A		
		Accounts Receivable Measures	Percentage of accounts receivable	Less than 0.11 per cent	—		0.11%→0.04%		—	
			The ratio of accounts receivable balance to medical revenue	Percentage Reduction	—		—		0.16%→0.16%→0.15%→0.15%→0.14%	
		Loan of assets held	Visiting nurse stations, etc.	Loan	—		—		7→9→12→13→15	
			Employment support business for persons with disabilities, etc.	Loan	—		—		5→5→6→7→8	
			University of Nursing Sciences	Loan	—		—		8→10→14→16→18	
		Obtaining research funding	Obtaining research funding	Increase in research expenses	(US$16.3M→US$24.5M)		US$24.5M→US$24.7M		Reclassified.	

Target item					Final review of medium-term					
Medium-term plan					1st		2nd		3rd	
Ⅲ. Matters Related to Improvement of Financial Status										
	Budget, Income and Expenditure Plan and Financial Plan				—	—	—	—		B
	Improvement of Management					S	US$352.7M→	A		B
		Achievement of profitability	Current account balance	Current account surpluses	Full-year achievement	S	US$352.7M→US$530.0M→US$416.4M→US$452.7M→US$288.1M	A	US$135.5M→US$7.3M→US$-61.8M→US$-20.0M→US$76.3M	B
		Achievement of profitability	total receipts and expenditure (disbursement)	US$316.4M→US$450.0M→US$-916.4M→US$380.9M→US$19.1M	US$316.4M→US$450.0M→US$-916.4M→US$380.9M→US$19.1M	S	US$316.4M→US$450.0M→US$-916.4M→US$380.9M→US$19.1M	A	US$106.4M→US$11.8M→US$-146.4M→US$-72.7M→US$16.4M	B
	Improvement of Fixed Liabilities Ratio					S	US$19.1M	S		B
		Investment	Total of Investment Limit; 1st: US$1.3B, 2nd: US$3.1B, 3rd: US$4.2B	Actual amount	(US$1.2B)	S	US$3.0B	S	US$3.0B	B
		Investment	Investment Limit for Medical Equipment; 1st: US$454.5M, 2nd: US$1.0B, 3rd: 1.4B	Actual amount	(US$637.3M)	S	US$1.2B	S	US$0.9B	B
		Investment	Investment Limit for Facility; 2nd: US$2.0B, 2rd: US$2.8B	Actual amount	—	S	US$1.7B	S	US$2.1B	B
		Fiscal Investment and Loan Borrowing; FILP system	Borrowing Amount	Actual value	—	S	US$580M	S	US$1.8B	B
		Fixed liabilities amount	Reduction of fixed liabilities	Reduction of fixed liabilities	US$6.8B→US$5.4B	S	US$5.4B→US$3.9B	S	US$3.9B→S$4.5B	B

Target item				Final review of medium-term					
Medium-term plan				1st		2nd		3rd	
IV. Other matters									
Other Matters Concerning Business Operation as Provided for in the Ordinance of the Competent Ministry									
	Registered nurse	Scholarship Loans	The number of loan recipients	(20→131)	A	(131→1,876)	A	745→822→782→710→613	B
			The number of loan recipients in employment	(14→53)		(53→761)		734→794→738→644→555	
	Employment of nursing students from affiliated nursing schools	Retention of nurses	Improvement of employment rate	56.7%→67.3%		—			
	Employment of the physically challenged	Employment of persons with disabilities	Achievement of legal employment rate (2.1%, after FY2013: 2.3%, after FY2018: 2.5%)	1.68%→2.42%		2.42%→2.11%		2.49%	
	Reduction of staff numbers	Reduction of technical staff	Non-supplementation	(1207)		(875)		543	
